# Rovibrational Spectroscopy of Trans and Cis Conformers of 2-Furfural from High-Resolution Fourier Transform and QCL Infrared Measurements

**DOI:** 10.3390/molecules28104165

**Published:** 2023-05-18

**Authors:** Sathapana Chawananon, Pierre Asselin, Jordan A. Claus, Manuel Goubet, Anthony Roucou, Robert Georges, Joanna Sobczuk, Colwyn Bracquart, Olivier Pirali, Arnaud Cuisset

**Affiliations:** 1 Sorbonne Université, MONARIS, CNRS, UMR8233, 4 Pl Jussieu, F-75005 Paris, France; sathapana.chawananon@sorbonne-universite.fr; 2 Université de Lille, CNRS, UMR8523—PhLAM—Physique des Lasers Atomes et Molécules, F-59000 Lille, France; jordan.claus@univ-lille.fr (J.A.C.); manuel.goubet@univ-lille.fr (M.G.); colwyn.bracquart@univ-littoral.fr (C.B.); 3 Université du Littoral Côte d’Opale, UR4493, LPCA, Laboratoire de Physico-Chimie de l’Atmosphère, F-59140 Dunkerque, France; anthony.roucou@univ-littoral.fr (A.R.); arnaud.cuisset@univ-littoral.fr (A.C.); 4 Université de Rennes, CNRS, IPR (Institut de Physique de Rennes)—UMR 6251, F-35000 Rennes, France; robert.georges@univ-rennes1.fr; 5 Smoluchowski Institute of Physics, Faculty of Physics, Astronomy and Applied Computer Science, Jagiellonian University, Łojasiewicza 11, 30-348 Kraków, Poland; joanna.b.dudek@doctoral.uj.edu.pl; 6 Université de Paris-Saclay, CNRS, Institut des Sciences Moléculaires d’Orsay, F-91405 Orsay, France; olivier.pirali@universite-paris-saclay.fr

**Keywords:** furfural, rovibrational spectroscopy, vibrational cross section, jet-cooling, QCL source, synchrotron-based FTIR spectroscopy

## Abstract

The ortho-isomer 2-furfural (2-FF), which is a primary atmospheric pollutant produced from biomass combustion, is also involved in oxidation processes leading to the formation of secondary organic aerosols. Its contribution to radiative forcing remains poorly understood. Thus, monitoring 2-FF directly in the atmosphere or in atmospheric simulation chambers to characterize its reactivity is merited. The present study reports an extensive jet-cooled rovibrational study of *trans* and *cis* conformers of 2-FF in the mid-IR region using two complementary setups: a continuous supersonic jet coupled to a high-resolution Fourier transform spectrometer on the IR beamline of the SOLEIL synchrotron (JET-AILES), and a pulsed jet coupled to a mid-IR tunable quantum cascade laser spectrometer (SPIRALES). Firstly, jet-cooled spectra recorded at rotational temperatures ranging between 20 and 50 K were exploited to derive reliable excited-state molecular parameters of *trans*- and *cis*-2-FF vibrational bands in the fingerprint region. The parameters were obtained from global fits of 11,376 and 3355 lines distributed over eight and three vibrational states (including the ground state), respectively, with a root mean square of 12 MHz. In a second step, the middle resolution spectrum of 2-FF recorded at 298.15 K and available in the HITRAN database was reconstructed by extrapolating the data derived from our low-temperature high-resolution analyses to determine the cross sections of each vibrational band of both 2-FF conformers in the 700–1800 cm^−1^ region. Finally, we clearly demonstrated that the contribution of hot bands observed in the room temperature 2-FF spectrum, estimated between 40 and 63% of the fundamental band, must be imperatively introduced in our simulation to correctly reproduce the HITRAN vibrational cross sections of 2-FF with a deviation smaller than 10%.

## 1. Introduction

Furfural (FF), also called furaldehyde (C_5_H_4_O_2_), is a furan derivative belonging to the family of oxygenated five-membered aromatic molecules. Identified as a primary and secondary pollutant in the atmosphere, it is emitted both by biogenic and industrial sources. FF is a reaction product of biomass combustion, which is an important source of trace substituents in the atmosphere. FF is a product of the pyrolysis of cellulose and the hemicellulose contained in the biomass [1]. Laboratory studies demonstrate that during combustion, high emission rates of FF are observed [2] and FF could be considered a marker volatile organic compound (VOC) for fire warning systems [3]. FF, which is readily produced from combustion, disappears rapidly as a result of atmospheric processes. The atmospheric oxidation of FF is likely to be initiated by photolysis or reactions with OH [4] and NO_3_ radicals [5], which are the two main oxidation processes during the day and night, respectively. These oxidation processes lead to the formation of secondary organic aerosols (SOA) that affect the climate via a direct or indirect contribution to radiative forcing, which remain poorly understood. FF is widely used in industry [6]; thus, most FF is emitted into the atmosphere by anthropogenic sources and its atmospheric oxidation leads to the formation of SOA and ozone cycle perturbation. For all these reasons, there is a strong interest in monitoring FF directly in the atmosphere or in atmospheric simulation chambers to identify its different sources and to characterize its reactivity and its ability to produce SOA.

Previous gas-phase spectroscopic investigations of FF mainly dealt with the ortho-isomer (2-FF) with vibrational analysis in the far-IR region using an infrared Fourier-transform interferometer (FTIR), in the mid-IR region using a Raman spectrophotometer [7], and in the VUV region using a synchrotron-based photoabsorption spectrometer [8]. Mid-IR vibrational cross sections are available in the HITRAN/PNNL atmospheric databases according to the medium resolution FTIR measurements (0.112 cm^−1^) of Johnson et al., who created a specific database for the quantitative IR spectroscopy of gases emitted by biomass burning [9]. In these studies, only the strong Q-branches and the P and R patterns were observed and no rovibrational analysis could be undertaken due to the limited spectral resolution. High-resolution analyses of FF have been performed in the microwave (MW) domain for 2-FF [10] and 3-FF [11] by means of jet-cooled Fourier transform microwave (FTMW) spectroscopy. Motiyenko et al. [10] extended their analysis in the millimeter-wave region at room temperature with the assignment and the analysis of the ground state (GS) spectrum for both *trans* and *cis* conformers and of the lowest energy torsional state for the most stable *trans*-2-FF only.

The present study aimed to determine the rovibrational parameters of both *trans* and *cis* conformers of 2-FF on a very broad IR domain. The present study is focused on the mid-IR region. Rovibrational line lists and high-resolution cross sections could be obtained for the most intense rotationally resolved vibrational bands. For the lowest energy rovibrational bands, the measurements performed at room temperature by means of synchrotron-based FT-Far-IR spectroscopy at the AILES beamline of the SOLEIL synchrotron facility using its high-resolution interferometer will be presented in an incoming paper. In order to reduce the rovibrational line density in the mid-IR due to hot band sequences, jet-cooled measurements were performed with two complementary experimental approaches [12]: broadband mid-IR measurements with the JET-AILES setup, and very accurate and sensitive measurements using two quantum cascade laser (QCL) sources centered at 6 and 10 μm coupled to the SPIRALES instrument.

## 2. Results

### 2.1. Vibrational Analysis

2-FF is an asymmetric top rotor close to the prolate limit (Ray’s parameter κ = −0.87) belonging to the $C_{s}$ point group of symmetry with 27 vibrational modes, of which 19 are in-plane of A’ symmetry and 8 are out-of-plane of A” symmetry. Modes are numbered according to Herzberg’s recommendation [13].

**Figure 1:**
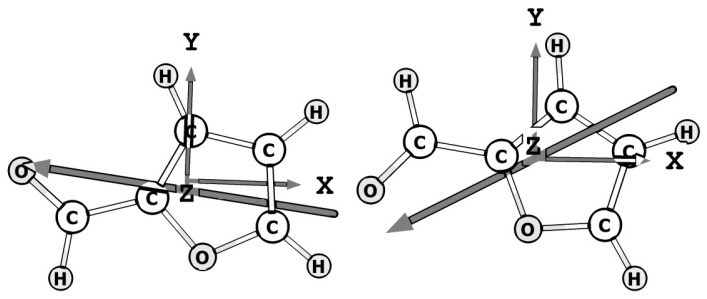
Calculated equilibrium geometry (hybrid/CBS) of the *trans-* and *cis*-2-FF conformers. The X,Y,Z axes correspond to the a,b,c principal axes, respectively. The large arrow indicates the orientation of the permanent dipole moment.

Previous spectroscopic far-IR, mid-IR, and Raman studies were performed at low resolution [7,14]. Durig et al. exploited far-IR and Raman data to estimate the relative stability between the *cis* and *trans* conformations of 2-FF in the gas phase (energy difference estimated to 3.42(29) kJ mol^−1^) and the conformational barrier height (38.94(24) kJ mol^−1^) from the modeling of an asymmetric torsional potential function [7]. On the grounds of branch separation of band contours and normal coordinate calculations, Adamek et al. assigned about 10 fundamental modes in the gas phase mid-IR spectrum of 2-FF and its deuterated analogue [14]. Taking advantage of its strong permanent dipole moment, with projections along the *a* axis of 3.20 D (3.41 D) and along the *b* axis of 0.40 D (1.93 D) for the *trans* (and *cis*) conformer (see Figure 1), Motiyenko et al. performed extensive microwave spectroscopic studies of 2-FF in both the centimeter- and millimeter-wave ranges [10,15]. The GS of both conformers and the first excited state (ES) of some low-frequency fundamental vibrational modes, namely, the ring-CHO torsion ($\nu_{27}$), the in-plane bending ($\nu_{19}$), and the out-of-plane bending ($\nu_{26}$) modes, were analyzed, providing molecular parameters of low-lying vibrational states up to 400 cm^−1^ within experimental accuracy. As expected for a high conformational barrier, no splitting due to the internal rotation of the CHO top was observed in either the ground or first torsional states ($\nu_{27}$).

**Figure 2:**
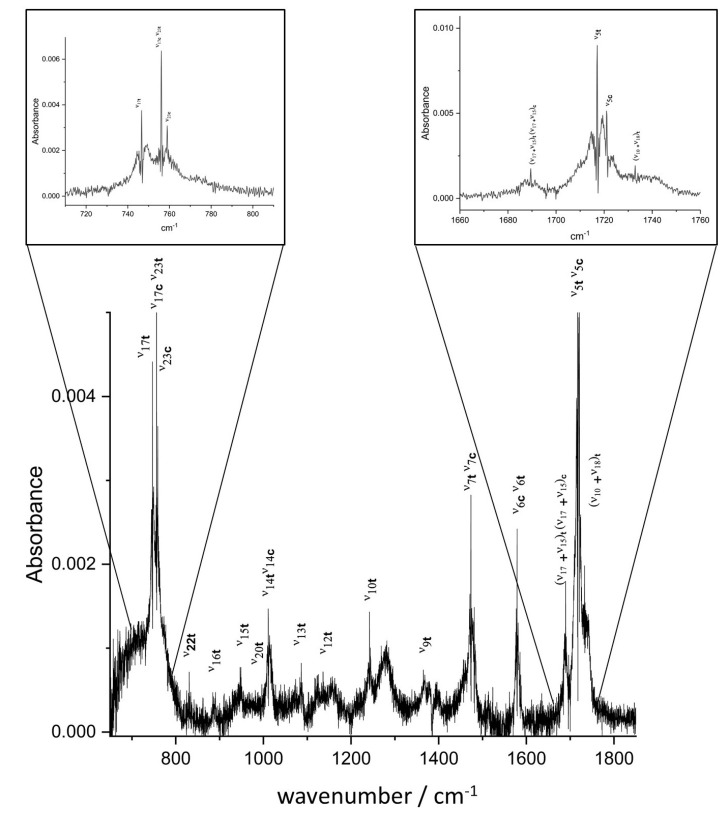
Jet−AILES FT-mid-IR spectrum of 2-FF measured at 0.5 cm^−1^ resolution displayed in the 730–1820 cm^−1^ region. Two insets are zoomed-in regions on the most intense bands. Vibrational assignments reported on the figure are based on comparison with hybrid/CBS anharmonic calculations. “t” and “c” in subscript indicate the “*trans*” and the “*cis*” conformers, respectively.

In the present work, most of the vibrational bands observed in the low-resolution (0.5 cm^−1^) FTIR spectrum recorded between 650 and 1850 cm^−1^ (see Figure 2) were clearly assigned on grounds of comparison with anharmonic frequency calculations performed both on the *trans* and *cis* conformers. The assignment was rather straightforward thanks to the good quality of the anharmonic prediction. For the *trans* conformer, most mid-IR fundamental bands were assigned and the strongest combination bands were also observed. For the *cis* conformer, which is higher in energy, the vibrational assignment was unambiguous for the most intense bands only, namely, $\nu_{14}$, $\nu_{7}$, $\nu_{6}$, $\nu_{17}+\nu_{15}$, and $\nu_{5}$. The complex assignment of close $\nu_{17}$ and $\nu_{23}$ bands of *cis*-2-FF in the 750 cm^−1^ region will be discussed in the following subsection. At high resolution, bands with calculated harmonic intensities larger than 50 km mol^−1^ were targeted to derive the excited-state molecular parameters from the rovibrational analysis of the jet-cooled spectrum at a 0.001 cm^−1^ resolution. Two band pass filters in the 650– 950 cm^−1^ and 1200– 1850 cm^−1^ ranges were used to cover the spectral range investigated. The full list of FTIR vibrational bands observed for both conformers is reported in Table 1 and compared to their calculated values at the anharmonic level. The targeted modes for the rovibrational analysis are associated with ring deformations in the ab plane ($\nu_{7}$ and $\nu_{6}$) and out-of-plane ($\nu_{23}$), an in-plane ring C-H bending ($\nu_{14}$) and a C=O stretching ($\nu_{5}$) and a C-C-H scissoring ($\nu_{17}$), involving the aldehyde group. In addition, the rovibrational analysis of the most intense ($\nu_{17}+\nu_{15}$) combination band could be performed.

### 2.2. Supersonic Jet Measurements

The complementarity between the highly sensitive but narrow bandwidth SPIRALES instrument and the very large bandwidth but less sensitive Jet-AILES setup is highlighted in the present high-resolution study: the Q branches identified in the Jet-AILES spectra over the fingerprint mid-IR region enabled us to evaluate which 2-FF rovibrational signatures can be reached with the SPIRALES setup covering the 9.7–10.2 μm and 5.8–6.3 μm ranges. Therefore, this section, which is dedicated to the rovibrational analysis, will be divided in three parts: first, the four bands that were intense enough for Jet-AILES but were outside of SPIRALES ranges, i.e., $\nu_{17}$ and $\nu_{23}$ around 750 cm^−1^, $\nu_{7}$ and $\nu_{6}$ in the 1450–1580 cm^−1^ range; second, two weak bands falling in SPIRALES ranges, i.e., $\nu_{14}$ and $\nu_{17}+\nu_{15}$; last, the $\nu_{5}$ band fully recorded up to 1726 cm^−1^ with Jet-AILES and partially with SPIRALES. In addition, the large difference in rovibrational cooling between Jet-AILES continuous and SPIRALES pulsed expansions leads to a lower rotational temperature with SPIRALES than with Jet-AILES, and consequently, gives access to different energy levels according to their respective rotational population distributions, in a complementary way.

All rovibrational analyses were initiated using the ES rotational constants from anharmonic calculations corrected from GS deviation.

#### 2.2.1. Jet-AILES Measurements: $\nu_{17},\nu_{23},\nu_{7}$, and $\nu_{6}$ Rovibrational Bands

At rotational temperatures (T${}_{rot}$) attained with Jet-AILES, typically around 50 K, the Doppler width of 2-FF in the 700–1750 cm^−1^ region ranges between 12 and 25 MHz (0.0004 and 0.0008 cm^−1^). In the 750 cm^−1^ region, Jet-AILES spectra recorded at 0.001 and 0.002 cm^−1^ resolution clearly indicate that line widths are only limited by the apparatus function; however, above 1450 cm^−1^, similar line widths were obtained at both resolutions. Consequently, $\nu_{17}$ and $\nu_{23}$ spectra were recorded at maximal resolution, while $\nu_{7}$, $\nu_{6}$, and $\nu_{5}$ were recorded at a 0.002 cm^−1^ resolution to maximize the signal-to-noise ratio (SNR).

**Figure 3:**
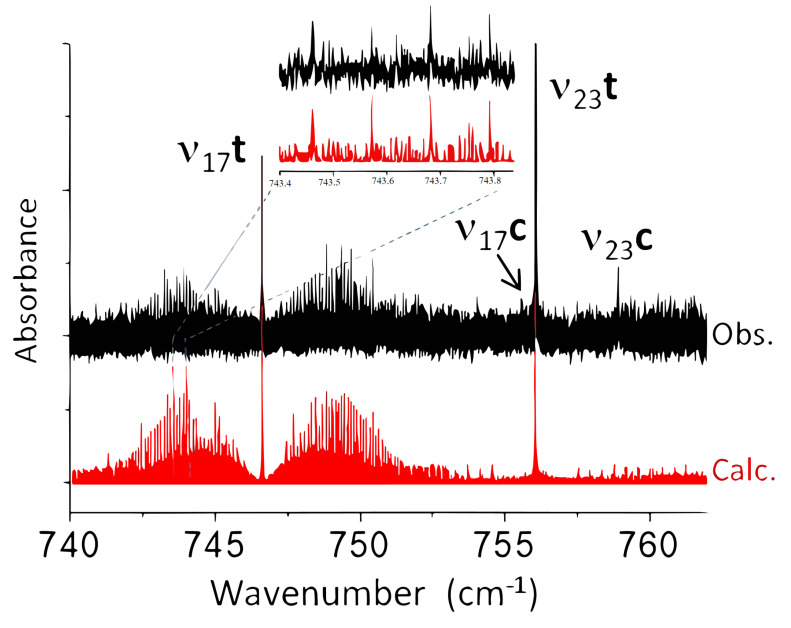
Overall view of $\nu_{17}$ and $\nu_{23}$ bands of both *trans* and *cis* conformers of 2-FF. In black, the Jet−AILES spectrum recorded at 0.001 cm^−1^ resolution. The intensity ratios of Q-branches assigned to these four bands correctly agree with the conformational energy difference determined by Durig et al. [7] for T${}_{vib}$ = 180(30)K. In red, the two *trans* conformer bands simulated at T${}_{rot}$ = 50 K. An expanded view in the P-branch of the $\nu_{17}$ band displays the good match between experimental and simulated spectra.

Figure 3 displays the Jet-AILES spectrum in the 740–762 cm^−1^ range where two characteristic band contours are observed: a Q-branch at 746.6 cm^−1^ surrounded by intense P and R branches typical of a *a*-type band, and a very intense Q-branch at 756.0 cm^−1^ surrounded by weak P and R branches typical of a *c*-type band. They were assigned to $\nu_{17}$ and $\nu_{23}$ bands of the *trans* conformer, respectively. This was in agreement with anharmonic calculations predicting $\nu_{17}$ to be about 5 cm^−1^ below $\nu_{23}$ and was confirmed by the orientation of the electric dipole moment, characteristic of a *c*-type band (A${}^{\prime \prime }$ symmetry) for $\nu_{23}$ and a *a/b* hybrid band (A${}^{\prime }$ symmetry) for $\nu_{17}$. The two weaker bands observed at 755.6 and 758.9 cm^−1^ should have corresponded to the $\nu_{17}$ and $\nu_{23}$ bands of the *cis* conformer, but anharmonic calculations failed to allow for an unambiguous assignment. Indeed, the difference between the two calculated frequencies of only about 1 cm^−1^ largely falls within the calculation uncertainty, so that assigning the 755.6 cm^−1^ band to $\nu_{17}$ and the 758.9 cm^−1^ band to $\nu_{23}$ (A) or vice versa (B) was possible. However, the intensity ratio between Q-branches of the two bands for each conformer measured in the Jet-AILES spectrum depends on the ratio of the energy difference between conformers and the vibrational states temperature (T${}_{vib}$) in the jet. The (B) assignment gave very different T${}_{vib}$ values (from 150 up to 400 K), which was unexpected for states of close energy and, moreover, higher than the reservoir temperature of the sample, while close T${}_{vib}$ values were obtained for both bands when choosing the (A) assignment.

**Figure 4:**
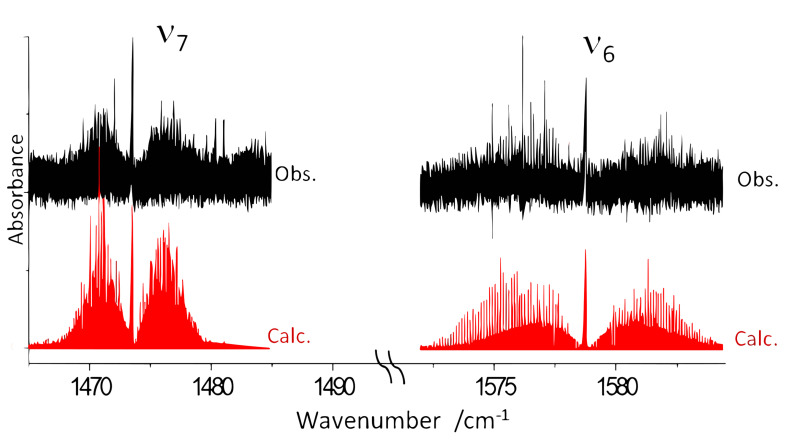
Overall view of the $\nu_{7}$ and $\nu_{6}$ band of *trans*-2-FF: in black the Jet−AILES spectrum recorded at 0.002 cm^−1^ resolution; in red, both bands simulated at T${}_{rot}$ = 50 K. The two structured Q-branches observed at 1480.3 and 1481 cm^−1^ are possibly involved in the perturbation of the $\nu_{7}$ band.

Figure 4 displays the Jet-AILES spectrum in both the 1465–1490 cm^−1^ and 1572–1585 cm^−1^ ranges of ring C=C asymmetric ($\nu_{6}$) and symmetric ($\nu_{7}$) stretching vibrations. The presence of two weak Q-branches (or one split Q branch) observed about 8 cm^−1^ higher than the band center of $\nu_{7}$ could be due to a rotational perturbation such as Coriolis or Fermi coupling.

#### 2.2.2. SPIRALES Measurements: $\nu_{14},\nu_{17}+\nu_{15}$ Rovibrational Bands

**Figure 5:**
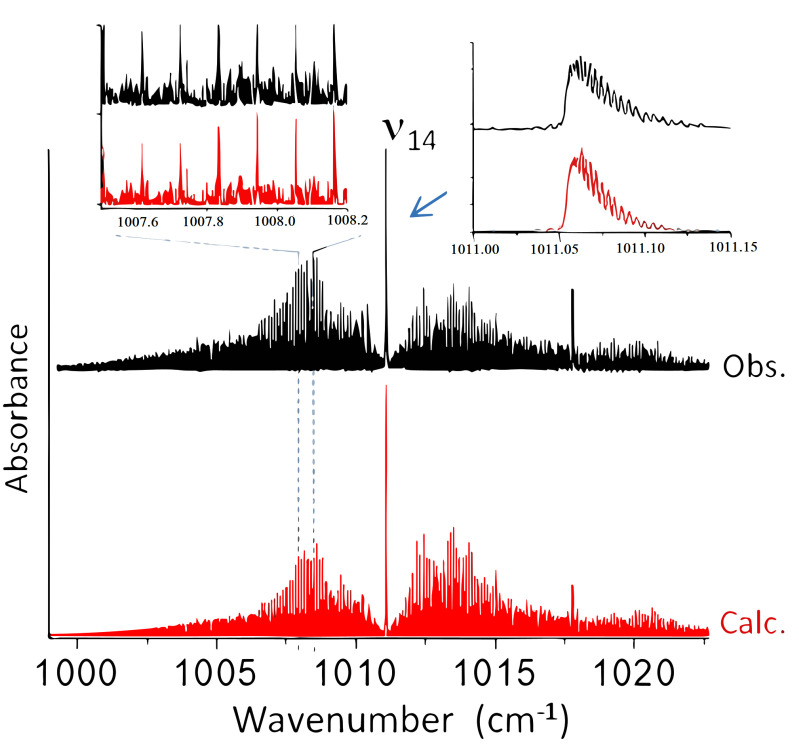
Overall view of the $\nu_{14}$ band of 2−FF: in black, the SPIRALES spectrum; in red, the $\nu_{14}$ band of *trans* and *cis* conformers simulated at T${}_{rot}$ = 30 K. Two expanded views of observed and calculated *trans* spectra in the P-Branch and the Q-branch are shown.

**Figure 6:**
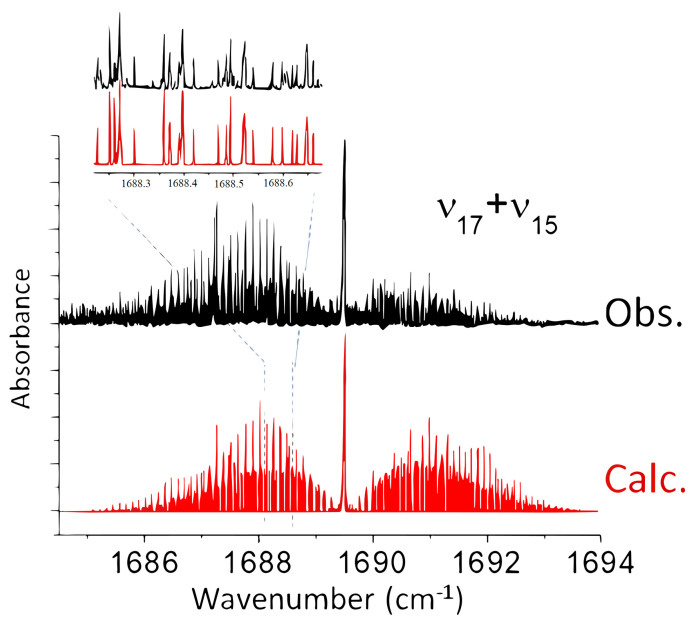
Overall view of the $\nu_{17}$ + $\nu_{15}$ combination band of *trans*−2−FF: in black, the SPIRALES spectrum; in red, the simulated spectrum at T${}_{rot}$ = 20 K. An expanded view of observed and calculated spectra in the P-branch is displayed.

Two fundamental bands of *trans*-2-FF, i.e., $\nu_{14}$ and $\nu_{5}$ calculated at 1017 and 1705 cm^−1^, fall within the range of our QCLs. Both bands were observed with the SPIRALES setup and an intense unexpected combination band $\nu_{17}+\nu_{15}$ predicted at 1700.5 cm^−1^ (13 km mol^−1^). The $\nu_{5}$ band measurements with the two jet-cooled setups will be detailed in the following section. Figure 5 displays the SPIRALES spectrum of the $\nu_{14}$ *a/b*-hybrid type band in both *trans* and *cis* conformers observed at 1011 and 1018 cm^−1^, respectively, about 5 cm^−1^ lower than the theoretical values. The $\nu_{17}+\nu_{15}$ combination band was observed with a poor SNR using Jet-AILES, while the more sensitive SPIRALES setup (see Figure 6) made it possible to perform the rovibrational analysis of the *trans* conformer band.

#### 2.2.3. SPIRALES and Jet-AILES Measurements: $\nu_{5}$ Rovibrational Bands

**Figure 7:**
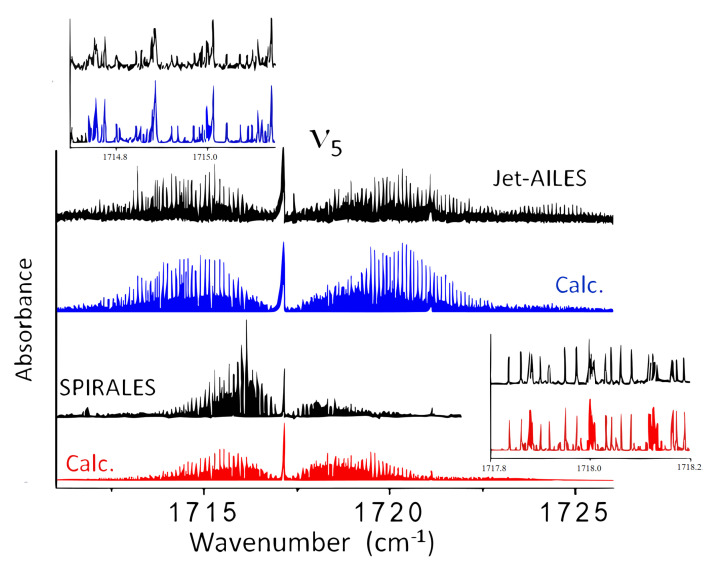
Overall view of the $\nu_{5}$ band of *trans-* and *cis*-2−FF: (top, black trace) the Jet-AILES spectrum; (middle, black trace) the SPIRALES spectrum; in blue, the simulated spectrum of both conformers at T${}_{rot}$ = 50 K with the Jet-AILES setup; in red, the simulated spectrum of both conformers at T${}_{rot}$ = 20 K with the SPIRALES setup. A relative abundance *trans*/*cis* equal to 3, similar to room temperature conditions, was assumed in the simulation. An expanded view of Jet-AILES and SPIRALES versus calculated spectra in the P- and R-Branches, respectively, is shown.

As announced above, Figure 7 displays the $\nu_{5}$ band measured with both jet setups at different rotational temperatures: the continuous supersonic flow of Jet-AILES imposed more concentrated 2-FF/Ar mixtures and lower backing pressures, typically between 100 and 300 hPa, leading to higher T${}_{rot}$ values than those achieved in the pulsed supersonic expansion of SPIRALES. The spectral range covered by SPIRALES did not enable us to record the *cis*-2-FF spectrum, while the full spectrum of the $\nu_{5}$ band recorded with Jet-AILES displays the band contour for both *trans* and *cis* conformers centered at 1717.1 and 1721.1 cm^−1^, respectively.

### 2.3. Rovibrational Analysis

Seven bands of *trans*-2-FF and two bands of *cis*-2-FF recorded at a high resolution in the mid-IR range in jet-cooled conditions were analyzed. Each band was firstly fitted individually with the PGOPHER program [16] using a Watson-type semirigid model for asymmetric tops (*a* reduction in the $I^{r}$ representation) developed up to the quartic centrifugal distortion (CD) terms. From the energy difference of Durig et al. between both conformers and our reservoir temperature ( 370 K), using a Boltzmann distribution, the sample before expansion was composed of 75% *trans*-2-FF and 25% *cis*-2-FF. It was difficult to assess precisely how it relaxed in the expansion but, in a high barrier approximation, we can assume that the ratio was roughly the same in the probed jet. Initially, GS parameters of both conformers were fixed to the values obtained by Motiyenko *et al*. For the *trans*-2-FF conformer, the $\nu_{17}$, $\nu_{23}$, $\nu_{14}$, $\nu_{17}+\nu_{15}$, and $\nu_{5}$ bands were recorded with a sufficient SNR so that band centers with rotational constants and most of the quartic CD constants could be adjusted. Due to the lower quality of the Jet-AILES spectrum for the weaker $\nu_{7}$ and $\nu_{6}$ bands and the presence of nearby vibrational states possibly responsible for anharmonic perturbation, only band centers and rotational constants were adjusted. For the *cis*-2-FF conformer, the small number of rovibrational lines only enabled us to assign a few quartic CD constants for the $\nu_{14}$ and $\nu_{5}$ bands. Finally, global fits were performed, including 1844 and 2488 GS rotational lines from the microwave study of Motiyenko et al. for *trans*- and *cis*-2-FF, respectively, and 9532 lines from seven vibrational states of *trans*-2-FF, ($v_{17}$ = 1, $v_{23}$ = 1, $v_{14}$ = 1, $v_{7}$ = 1, $v_{6}$ = 1, $v_{17},v_{15}$ = 1,1 and $v_{5}$ = 1) and 867 lines from two vibrational states of *cis*-2-FF, ($v_{14}$ = 1, $v_{5}$ = 1). A total of 11,376 lines for *trans*-2-FF and 3355 lines for *cis*-2-FF were fitted to instrumental accuracy with root mean square (RMS) values of 0.00039 and 0.00037 cm^−1^ for *trans*- and *cis*-2-FF, respectively. The upper state rotational parameters of both conformers are reported in Table 2 and Table 3. Tables including line assignments, measured frequencies, uncertainties, and deviations are provided as the Supplementary Material.

## 3. Discussion

### 3.1. Comparison between Theoretical and High-Resolution Experimental Results

The GS- and ES-calculated rotational constants at the hybrid/CBS level are compared to the experimental values for both 2-FF conformers in Table 4. The mean absolute error (MAE) of the differences (δ=exp−calc) between the experimental and calculated rotational constants are equal to 6.4 MHz for *trans*-2-FF and 2.1 MHz for *cis*-2-FF over 24 and 9 constants, respectively. After correction of the calculated values using the GS deviation (see Equation (2)), the MAEs of the corrected values decreased to 3.3 MHz for *trans*-2-FF and 0.7 MHz for *cis*-2-FF, mainly due to the larger absolute value of the A rotational constant resulting in a larger deviation. Indeed, the MAE for *trans*-2-FF was considerably reduced to only 630 kHz by considering only the B and C corrected constants (14 ES), which gives good confidence in the predictive power of these corrected constants, as was previously shown for similar systems [17,18,19].

In Table 4, we also calculated the second (or planar) moments $M_{cc}$, which took into account the displacement of the masses along the c–axis perpendicular to the ab plane of *trans*-2-FF and *cis*-2-FF. This second moment must be equal to 0 for a perfectly planar molecule [20]. This statement was checked for all the $M_{cc}$ values determined in Table 4 ($|M_{cc}|<0.1$), except for the $\nu_{23}$ and $\nu_{7}$ of *trans*-2-FF. In the case of $\nu_{23}$, we expected a larger inertial defect induced by the loop nature of the vibration. For the $\nu_{7}$ band, the larger $M_{cc}$ experimental value was unexpected since the $\nu_{7}$ is an ip ring mode and the $M_{cc}$ calculated value was predicted close to 0. Such an anomaly may again be a signature of a rotational perturbation (Coriolis or Fermi coupling) as already mentioned in Section 2.2.1. Except for $\nu_{6}$, the δ values were systematically positive, suggesting a contribution of the zero-point vibrational motion to the non-planarity. This was also confirmed by the decrease in the δ values when we corrected the calculated rotational constants from the GS deviation.

As already mentioned, the predictions in [17] were less accurate for vibrational frequencies than for rotational constants. Although far from being satisfactory for high-resolution purposes, rough predictions are sufficient to assign observed bands. A total of 23 experimental frequencies were observed and assigned (see Table 1), based on predictions with a MAE of the differences between the experimental and calculated values of 10 cm^−1^, which corresponds to a mean relative error of 0.8%. In particular, $\nu_{9}$ was the most poorly predicted, with an estimation at almost −33 cm^−1^. Such a large deviation might have been due to either calculations errors or an anharmonic resonance between these two bands. All other bands were predicted at 1.9% or less, permitting unambiguous assignments. Concerning combination bands, they were assigned from a list of selected examples predicted at ±30 cm^−1^; according to their clear *a/b* hybrid band shape, A” symmetry bands were excluded; then, within the remaining examples, the calculated anharmonic intensities clearly pointed toward the assigned examples.

### 3.2. Mid-IR Cross Sections

Two infrared spectra of FF were measured by Johnson et al. in the 550–6500 cm^−1^ spectral range [9]. These spectra, which are available in the HITRAN atmospheric database [21], were measured using FTIR spectroscopy in a systematic study of biomass burning compounds with a resolution of 0.112 cm^−1^ at 760 Torr in N${}_{2}$, at 298.15 K, and at 323.15 K. Medium-resolution vibrational cross sections were deduced for quantitative spectroscopy applications. In this section, we extrapolated the data obtained from our low-temperature high-resolution analysis to understand and predict these mid-IR cross sections at 298.15 K.

**Figure 8:**
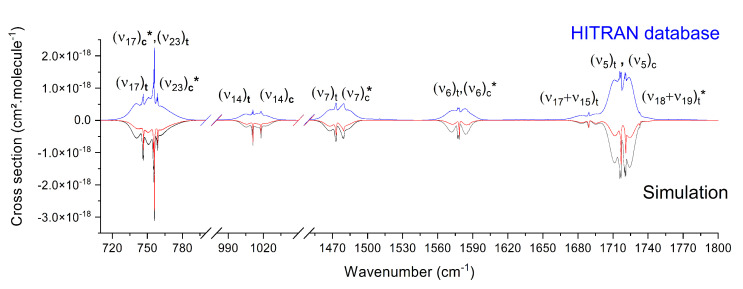

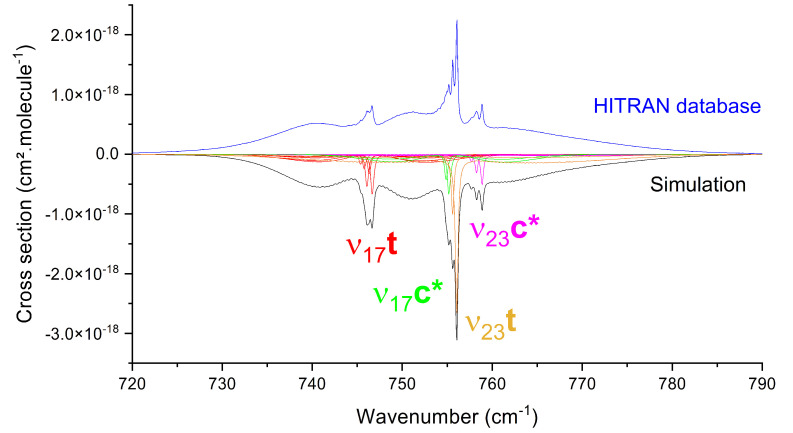
**Top**: Comparison of 2-FF room temperature integrated cross sections from the HITRAN database (blue line) and band contour PGOPHER simulations using jet-cooled high-resolution data (red line). The simulated band contours were constructed with the fitted parameters summarized in Table 2 and Table 3 from the nine rovibrational bands analyzed at high resolution. Five additional bands (marked with a star) were added with molecular parameters scaled from the fitted ones of the *trans* conformer $B_{vib}^{cis}=\frac{B_{GS}^{cis}\times B_{vib}^{trans}}{B_{GS}^{trans}}$, except for the $\left(\nu_{18}+\nu_{10}\right)$ combination band which was not analyzed at high resolution for the *trans* conformer. The addition of hot bands was required to reproduce the cross sections measured at room temperature (black line). **Bottom**: Expanded view of the HITRAN cross sections of both $\nu_{17}$ and $\nu_{23}$ bands (blue line) compared to simulations of fundamental and hot band contours of *trans*- and *cis*-2-FF conformers (black line for the full simulation; red, green, orange, and magenta for the ${\left(\nu_{17}\right)}_{t}$, ${\left(\nu_{17}\right)}_{c}$, ${\left(\nu_{23}\right)}_{t}$, and ${\left(\nu_{23}\right)}_{c}$ individual contributions, respectively).

First of all, we reconstructed the cross sections of each vibrational band of the *trans* and *cis* conformers including the parameters of our global fit summarized in Table 2 and Table 3. The results are shown in Figure 8 (top, red curve). The intensities of each band, depending on both the relative abundance *trans*/*cis* and dipole moment value were adjusted individually to reproduce the experimental cross sections to the best of our ability. Noticeably, the presence of many hot bands should be considered, in particular, for transitions starting from low-frequency vibrational states (hereafter, designated by lf) up to 600 cm^−1^, as each of them contributes more than 5% of the fundamental band intensity at room temperature. In the first step, the contribution of hot bands, for which Q branches were clearly visible in the room temperature spectrum, was added. The positions and the intensities of these hot bands were adjusted to optimize the agreement with the database cross sections. For the simulation, we considered the hot band rotational constants equal to those of the associated cold band. We kept the same resolution as the HITRAN cross sections and chose collisional broadened Lorentzian profiles with a FWHM set to 0.3 cm^−1^.

The simulations including hot bands are shown in Figure 8 (top, black curve). Our results gathered in the Table 5 exhibit a correct agreement between the HITRAN-integrated cross sections over the spectral windows of the fingerprint region, namely, 700–795, 980–1050, 1450–1550, 1550–1610, and 1660–1800 cm^−1^. All the deviations were lower than 10%, except in the 1450–1550 cm^−1^ range belonging to the $\nu_{7}$ mode, where deviations amounted to 25%, possibly due to the presence of anharmonic couplings. The comparison between simulations with and without hot bands shows that, at room temperature, hot bands contributed from 40% to 63% to the full cross section. An accurate model of these hot bands is required to perfectly simulate the vibrational cross sections. The position of these hot bands allowed us to make a preliminary estimation of the $\chi_{i,j}$ anharmonic coefficients. The detailed simulation with the PGOPHER software [16] of the $\nu_{17}$ and $\nu_{23}$ bands around 750 cm^−1^ is given as example in Figure 8 (bottom). It includes the asymmetric top band contour with $J_{max}$ = 140 of four fundamental hot bands: ${\left(\nu_{17}\right)}_{t}$, ${\left(\nu_{17}\right)}_{c}$, ${\left(\nu_{23}\right)}_{t}$, and ${\left(\nu_{23}\right)}_{c}$, and eight additional hot bands: four hot bands for ${\left(\nu_{17}\right)}_{t}$ with two sequences with $\chi_{17t,lf1t}$ = −0.6 cm^−1^ and $\chi_{17t,lf2t}$= −0.35 cm^−1^; one for ${\left(\nu_{17}\right)}_{c}$ with $\chi_{17c,lf1c}$ = −0.3 cm^−1^; one for ${\left(\nu_{23}\right)}_{t}$ with $\chi_{23t,lf1t}$ = −0.45 cm^−1^; two for ${\left(\nu_{23}\right)}_{c}$ with $\chi_{23c,lf1c}$ = −0.6 cm^−1^. lf1 and lf2 denote two low-frequency modes susceptible to being sufficiently populated at room temperature and to being the starting vibrational energy levels involved in the observed hot bands. Due to lack of accuracy of the calculated $\chi_{i,j}$ anharmonic coefficients, it was impossible to unambiguously assign the lf1 and lf2 modes by considering the theoretical values. Nevertheless, a quick overview of the hot bands observed next to the other mid-IR bands analyzed in the fingerprint region revealed some tendencies: a first hot band sequence composed of one or two members, with $\chi_{i,j}$ between −0.4 cm^−1^ and −0.7 cm^−1^ red-shifted from the fundamental band, and sometimes a second one with $\chi_{i,j}$ close to −0.4 cm^−1^ also red-shifted. Moreover, we noted that the magnitudes of these anharmonicity coefficients were both mode- and conformer-dependent as observed for both the $\nu_{14}$ and $\nu_{5}$ modes. On the grounds of only the jet-cooled mid-IR high-resolution and low-temperature spectra, we cannot propose any convincing assignment of the low-frequency modes involved in the hot band sequences. In the far-IR study of 2-FF performed in Ref. [7], the lowest energy vibrations show large sequences of hot bands, again red-shifted from their origin. In particular, the lowest energy mode $\nu_{27}$ associated with the ring-CHO torsion presents a sequence up to $6\nu_{27}\leftarrow 5\nu_{27}$ for the *trans* conformer, allowing an accurate determination of $\chi_{27,27}=-0.48\;\pm \;$ 0.03 cm^−1^ ($\chi_{27,27}$ = −0.2 cm^−1^ for the *cis* conformer). Red-shifted hot band sequences were also clearly observed for the other low-frequency bending modes of the aldehyde group: the rocking $\nu_{19}$ and the twisting $\nu_{26}$ centered at 202.5 cm^−1^ and 237 cm^−1^, respectively, for the *trans* conformer. At room temperature, these three low-frequency modes were sufficiently populated to be involved in the hot bands observed in the mid-IR range spectra discussed in the present work. Their assignment and the determination of the $\chi_{i,j}$ off-diagonal anharmonic coefficients (with j = 19, 26 and 27) require accurate anharmonic calculations involving sophisticated variational procedures [22] and a high-resolution analysis of the room temperature long pathlength cell far-IR spectrum [12]. This work is under progress and will be the subject of a future publication.

## 4. Materials and Methods

### 4.1. Theoretical Methods

Calculations were performed using the Gaussian 16 rev. C.01 software [23] on the computing clusters of the PhLAM laboratory. The frozen-core approximation was used throughout. Dunning and coworkers’ augmented correlation consistent basis set aug-cc-pVXZ (X = D, T, Q) was used (denoted aVDZ, aVTZ, and aVQZ) [24]. All geometries were fully optimized at the MP2 and B98 levels using the tight convergence criterion. Extrapolations to complete the basis set (CBS) for energies (including ZPE corrections), band centers, and rotational constants were performed from aVDZ, aVTZ, and aVQZ results using Dunning’s formula [25]. Frequencies and rotational constants in relevant vibrational states were calculated at the anharmonic level (VPT2 calculations as implemented in the Gaussian software) [26] with a tight SCF convergence criterion and the ultra-fine integral grid option. Anharmonic corrections were extrapolated from DFT (B98) to the MP2 level following a method (denoted “hybrid”) suggested by Barone et al. [27], which was successful in the case of systems containing carbonyl groups [18,28,29]. Briefly, since anharmonic calculations at the MP2 level are hardly affordable for such a relatively large molecule, the energies, band centers, and rotational constants of a given vibrational state *v* (called “hybrid”) are estimated by adding DFT anharmonicity (B98/CBS) to the MP2 constants at equilibrium eq (MP2/CBS). For example:

(1) $$B_{v}^{hybrid}=B_{eq}^{MP2}-\left(B_{eq}^{B98}-B_{v}^{B98}\right)$$

where $B_{v}$ is the rotational constant of the vibrational state *v*, and $B_{eq}$ is the rotational constant at equilibrium. In cases where CBS convergence is not satisfactory (i.e., aVDZ, aVTZ, and aVQZ points do not show a correct exponential shape), values from the highest level (aVQZ) are used instead.

Concerning rotational constants, once experimental GS values are known, calculated ES rotational constants values can be corrected from the GS deviation:

(2) $$B_{v}^{corr}=\frac{B_{v}^{calc}\times B_{GS}^{exp}}{B_{GS}^{calc}}$$

### 4.2. JET-AILES

The Jet-AILES setup was described in detail in previous works [30]. The continuous supersonic expansion is generated by a heatable 81 mm long slit nozzle in a vacuum chamber evacuated by one primary rotary pump and two secondary root pumps delivering a pumping speed of 1850 $m^{3}$ h^−1^. Total gas flow rates up to 40 slm can be achieved with a maximal residual pressure of about 1 hPa. The stagnation pressure can be adjusted up to 3000 hPa by changing the flow rate or by changing the width of the slit nozzle from 20 to 250 μm, which is estimated from the stagnation pressure by assuming an inviscid flow and sonic conditions (i.e., Mach number of 1) at the nozzle exit. The high-pressure reservoir and the low-pressure vacuum chamber are equipped with baratron pressure gauges. The injected gases are regulated with a series of mass-flow controllers (Bronkhorst). The liquid FF sample is evaporated in a controlled manner using a controlled evaporation mixer (CEM, Bronkhorst) supplied with regulated flows of argon as buffer gas and liquid FF. In the present study, the slit nozzle, the gas supply line, and the CEM were maintained at 400 K to avoid any recondensation of the sample downstream the CEM and to prevent the temperature of the nozzle from dropping down due to the supersonic jet cooling.

The vacuum chamber is connected to the high-resolution Bruker IFS 125 FTIR spectrometer installed on the AILES beamline of the SOLEIL synchrotron facility. The present jet experiments rely on the spectrometer’s internal mid-infrared source (globar). After being modulated, the infrared light beam is focused at about 2 mm from the exit of the planar supersonic expansion and collected by a liquid nitrogen cooled HgCdTe detector. The vacuum chamber is isolated from the spectrometer and the detectors compartment with two ICS windows placed on either side of the supersonic expansion. A KBr beamsplitter is used to record spectra in the 600–1800 cm^−1^ spectral range. A series of 200 low-resolution (0.1 cm^−1^) reference spectra are systematically recorded before injection of FF in order to calculate the transmittance spectra.

The optimization of supersonic expansion parameters, such as argon and FF flows, the stagnation pressure, and the concentration ratio sample/argon of Jet-AILES enabled us to optimize the signal-to-noise ratio (SNR) of vibrational signatures. The optimized parameters used to record spectra with this setup can be found in Table 6. The choice of argon instead of helium as carrier gas was justified by its ability to lower the rotational temperature due to a better efficiency of Ar-FF binary collisions in terms of energy transfer compared to He-FF [12]. As a consequence, the intensity of colder rovibrational lines is increased thanks to a narrower rotational distribution. In the present study, the stagnation pressure was limited to 320 hPa or even less to prevent argon clustering. Indeed, argon pressure that too high, however, favors the formation of $Ar_{n}$ heterocomplexes, leading to characteristic broad and unstructured absorption features red-shifted from the monomer absorption bands, and a concomitant reduction in the intensity of the monomer absorption bands. It should be noted that the widths reported in Table 6 are effective and slightly underestimated because the assumption of an inviscid gas does not take into account the boundary layers that form on the walls of the nozzle, which increase the stagnation pressure by slowing down the gas flow.

A low-resolution spectrum (0.5 cm^−1^, condition #1) was first recorded to locate the absorption features of 2-FF between 650 and 1800 cm^−1^. The jet-cooled spectrum in the fingerprint region displayed in Figure 2 results from the Fourier transform of 90 co-added interferograms at a 0.5 cm^−1^ resolution, recorded with 0.5 slm of 2-FF diluted in 5 slm of argon. Two high-resolution spectra were then recorded at the maximum resolution of the spectrometer equipping the AILES beamline (0.00102 cm^−1^, conditions #2 and #3) using 1 slm of 2-FF diluted in 10 slm of argon. Band pass filters were used to optimize the SNR in two distinct spectral regions centered at about 800 cm^−1^ (see Figure 3) and 1500 cm^−1^ (see Figure 4 and Figure 7), respectively.

### 4.3. SPIRALES

IR direct laser absorption experiments on 2-FF were performed with a jet-cooled laser spectrometer (hereafter, named the SPIRALES setup), which couples an external-cavity quantum cascade laser (EC-QCL) and a pulsed supersonic free jet to probe gas phase molecules cooled in the adiabatic expansion. SPIRALES was described in details in recent papers [31,32], and only the main characteristics and most recent developments are presented hereafter.

The IR source is a continuous-wave room-temperature mode-hop-free EC-QCL (Daylight Solutions) of 10 MHz spectral width. The QCL chip and a diffraction grating are mounted on a piezoelectric transducer (PT) to form an external cavity, and high-resolution measurements are obtained by scanning the length of this cavity. In the present study, two EC-QCLs were used to cover the following spectral ranges: 975–1035 cm^−1^ (Model 41103-MHF) and 1620–1720 cm^−1^ (Model 21060-MHF). About 8% of the total power is used by an etalon consisting of a 0.025 cm^−1^ free-spectral-range confocal Fabry–Perot cavity, to provide a relative frequency scale. Absolute frequency calibration is obtained by passing about 8% of the IR total radiation through a 10 cm length cell containing a known reference gas. A linear interpolation of the positions of the etalon maxima establishes the relationship between the voltage applied to the PT and the relative frequency. This new frequency scale enables one to correct the free-spectral-range value of the reference fixed at the beginning of each experiment. A typical frequency accuracy of about 0.0005 cm^−1^ was achieved by comparing the frequency deviation of our measured lines of methanol (in the 975–1035 cm^−1^ range) and NH${}_{3}$ and H${}_{2}$O (in the 1620–1720 cm^−1^ range) with frequency standards from the HITRAN2020 database [33]. About 85% of the initial laser power is directed toward a multi-pass absorption cavity, based on an astigmatic variant of the off-axis resonator Herriott configuration. This optical cavity composed of two 1.5 inch astigmatic mirrors (R = 99.2%, AMAC-36, Aerodyne Research) is installed in the supersonic expansion chamber, perpendicularly to the jet axis. With respect to the square spot pattern of the initial optical configuration adjusted for 182 passes, the present optical settings were modified to obtain a rectangular spot pattern, which overlapped better with the planar expansion but with about half of the optimum number of optical passes.

With the QCL setup, pulsed planar expansions synchronized with a laser wavelength sweep and the simple implementation of multi-pass optical cavities make it possible to work at large backing pressures with very diluted M/Rg samples, where M is the molecule studied and Rg is a rare gas. Previous studies [34] showed that M-Rg van der Waals heterodimers could be typically formed in the following conditions: 1–2% M diluted in 4000 hPa Rg and 4000 hPa He. In the present 2-FF study, however, the pulsed jet conditions used with the QCL setup (1% 2-FF in 2000 hPa Ar) were rather well adapted to efficiently cool down the rotational temperature of 2-FF and to drastically reduce hot bands without forming Ar heteroclusters because the backing pressure was kept relatively low. The molecular jet was produced from a pulsed 0.9 mm diameter pin hole nozzle from General Valve Series 9 controlled by a valve driver (Iota One, Parker Hannifin). FF compounds were seeded in the supersonic jet using a brass block fitted to a Dural reservoir filled with 1 g of liquid sample. The reservoir located upstream, nearby the expansion zone, was heated up to 370 K to increase the sample vapor pressure, which was carried by the argon flow. The seeded mixture was then cooled down by converting the circular flow of the standard valve configuration into a planar expansion using six-way distribution gas channel capped with two modified industrial blades, forming a 30 mm length and 150 μm width slit aperture. Jet-cooled FF molecules were probed over axial distances between 5 and 15 mm from the nozzle exit due to the relatively large zone covered by the different trajectories of the IR beam in the multi-pass optical system. Jet-cooled spectra were recorded using a rapid scan scheme similar to setups developed previously and described in Ref. [35]. The QCL frequency was scanned by a sine wave with an amplitude of up to 80 V to the PT at frequencies up to 100 Hz, which corresponds to a sweep of 0.8 cm^−1^ in 5 ms with a frequency sampling of about 3 MHz. The operating frequency of the pulsed valve was typically equal to 1 Hz. A baseline-free transmittance through the multi-pass cavity was obtained by taking the ratio of signals recorded in the presence and absence of the jet.

## 5. Conclusions

The association of two jet-cooled mid-IR high-resolution spectroscopies, one based on a synchrotron source (the JET-AILES setup) and the other one based on QCL sources (the SPIRALES setup), complemented with quantum-chemistry anharmonic calculations allowed us to measure, resolve, and assign seven rovibrational bands for the *trans* and two additional bands for the *cis* conformer of 2-FF. The molecular parameters in these excited rovibrational states of the fingerprint region and the ground-state parameters were globally fitted from 11,376 *trans* and 3355 *cis* experimental rovibrational lines. These parameters allow us to reproduce the mid-IR spectra at the experimental accuracy and may be used to reconstruct the vibrational cross sections used for quantitative spectroscopy in the atmosphere by extrapolation. In particular, we tried to reproduce the vibrational cross sections referenced in the HITRAN atmospheric database measured at room temperature in a N${}_{2}$ dilution. Using this approach, we clearly demonstrated the importance of hot bands in the room-temperature mid-IR spectra of 2-FF, which contribute between 40% and 63% of the fundamental bands. Experimental values presented here can be used to calibrate higher level calculations in order to obtain more accurate predictions of the anharmonic constants. Indeed, this work highlighted the necessity of a reliable anharmonic force field that is able to provide sufficiently accurate anharmonic coefficients $\chi_{i,j}$, allowing the hot band pattern to be assigned and the reconstruction of the room-temperature rovibrational cross sections to be improved for this kind of medium-sized VOCs, for which it is not possible to resolve the rovibrational structure at room temperature.

## Figures and Tables

**Table 1:** 2-FF vibrational mid-IR assignments based on experimental measurements and anharmonic calculations at the hybrid/CBS level. All vibrational frequencies are given in cm${}^{-1}$ unit.

$\nu_{\exp}$	Mode	Symmetry	Conformer	$\nu_{\mathrm{harm}}$	$\nu_{\mathrm{anharm}}$	Description
1732.9	$\nu_{18}+\nu_{10}$	A’	trans	1779.6	1755.4	
1721.1	$\nu_{5}$	A’	cis	1732.5	1705.3	C=O stretching
1717.1	$\nu_{5}$	A’	trans	1726.4	1704.8	C=O stretching
1689.5	$\nu_{17}+\nu_{15}$	A’	trans	1719.4	1700.5	
1687.2	$\nu_{17}+\nu_{15}$	A’	cis	1722.2	1692.1	
1578.8	$\nu_{6}$	A’	trans	1595.0	1566.1 *	ring C=C asym stretching
1577.1	$\nu_{6}$	A’	cis	1584.4	1554.2 *	ring C=C asym stretching
1481.1	$\nu_{7}$	A’	cis	1506.8	1479.2 *	ring C=C sym stretching
1473.6	$\nu_{7}$	A’	trans	1500.1	1475.8 *	ring C=C sym stretching
1365.0	$\nu_{9}$	A’	trans	1402.1	1368.8	C-H ip bending
1242.1	$\nu_{10}$	A’	trans	1281.7	1259.0	ring C-H ip bending
1136.0	$\nu_{12}$	A’	trans	1193.6	1173.3	ring C-H ip bending
1086.4	$\nu_{13}$	A’	trans	1116.2	1101.8	ring C-H ip bending
1017.7	$\nu_{14}$	A’	cis	1044.3	1022.7 *	ring C-H ip bending
1011.0	$\nu_{14}$	A’	trans	1038.6	1016.9 *	ring C-H ip bending
948.6	$\nu_{15}$	A’	trans	963.7	952.4	ring ip bending
886.9	$\nu_{16}$	A’	trans	896.1	888.2	ring ip bending
755.6	$\nu_{17}$	A’	cis	769.7	759.2	C-C-H scissoring
746.6	$\nu_{17}$	A’	trans	755.7	750.6	C-C-H scissoring
996.4	$\nu_{20}$	A”	trans	1010.5	996.7 *	C-H oop bending
830.6	$\nu_{22}$	A”	trans	840.9	814.6 *	ring C-H oop bending
758.9	$\nu_{23}$	A”	cis	775.5	757.9 *	ring C-H oop bending
756.1	$\nu_{23}$	A”	trans	769.2	755.2 *	ring C-H oop bending

* no CBS convergence of anharmonic frequencies: hybrid values calculated using B98/aVQZ anharmonicity.

**Table 2:** Molecular parameters (in MHz) of the ground state, $v_{17}$ = 1, $v_{23}$ = 1, $v_{14}$ = 1, $v_{7}$ = 1, $v_{6}$ = 1, $v_{17},v_{15}$ = 1,1, and $v_{5}$ = 1 of *trans*-2-FF derived from the combined fit of eight states.

Frequency	GS	$\nu_{17}$	$\nu_{23}$	$\nu_{14}$	$\nu_{7}$	$\nu_{6}$	$\nu_{17}+\nu_{15}$	$\nu_{5}$
(cm${}^{-1}$)		746.59621(2)	756.05273(2)	1011.05284(2)	1473.58372(3)	1578.77264(3)	1689.52004(2)	1717.14825(2)
A	8191.77383(13)	8196.9940(13)	8178.0125(220)	8196.3949(148)	8168.443(32)	8184.040(38)	8181.784(119)	8179.2141(305)
B	2045.929569(13)	2045.2823(58)	2045.3409(170)	2046.3469(48)	2041.9928(86)	2044.0863(87)	2043.6672(124)	2043.8979(101)
C	1637.183877(12)	1636.2156(47)	1637.5243(238)	1637.2606(37)	1636.8828(54)	1635.6319(25)	1635.0497(96)	1635.5041(61)
$\Delta_{J}\left(\times 10^{3}\right)$	0.1361873(39)	0.1342(20)	0.155(8)	0.1379(18)	0.1361873	0.1361873	0.110(8)	0.1269(41)
$\Delta_{K}\left(\times 10^{3}\right)$	1.7829(24)	1.7316(21)	2.114(111)	2.694(45)	1.7829 ${}^{a}$	1.7829 ${}^{a}$	−0 .0456(26)	2.771(135)
$\Delta_{JK}\left(\times 10^{3}\right)$	0.706618(18)	0.9057(13)	0.808(57)	0.875(16)	0.706618 ${}^{a}$	0.706618 ${}^{a}$	3.59(32)	0.642(44)
$\delta_{J}\left(\times 10^{3}\right)$	0.0314688(12)	0.0306(14)	0.0418(122)	0.0283(12)	0.0314688 ${}^{a}$	0.0314688 ${}^{a}$	0.0314688 ${}^{a}$	0.0469(32)
$\delta_{K}\left(\times 10^{3}\right)$	0.82192(118)	0.544(82)	0.82192 ${}^{a}$	0.877(75)	0.82192 ${}^{a}$	0.82192 ${}^{a}$	0.82192 ${}^{a}$	0.82192 ${}^{a}$
$\Phi_{J}\left(\times 10^{9}\right)$	0.01758(44)							
$\Phi_{JK}\left(\times 10^{9}\right)$	0.6571(32)							
$\Phi_{K}\left(\times 10^{9}\right)$	5.097(13)							
IR RMS	0.0099	10.8	9.9	17.4	18.9	24.6	15.9	13.8
N lines	1844	2401	1224	3446	513	508	662	999
$J^{\prime \prime }$	1–99	1–56	1–39	1–56	2–33	4–52	4–52	1–52
$K_{a}^{\prime \prime }$	0–53	0–35	0-21	0–22	0–14	0–14	0–10	0–20

${}^{a}$ Fixed to the GS value.

**Table 3:** Molecular parameters (in MHz) of the ground state, $v_{14}$ = 1, and $v_{5}$ = 1 of *cis*-2-FF derived from the combined fit of three states.

Frequency	GS	$\nu_{14}$	$\nu_{5}$
(cm${}^{-1})$		1017.76832(2)	1721.12234(3)
A	8143.738729(71)	8149.490(40)	8135.273(67)
B	2098.724250(14)	2099.3461(62)	2096.7713(99)
C	1668.872904(14)	1668.9979(58)	1667.4017(61)
$\Delta_{J}\left(\times 10^{3}\right)$	0.1726591(62)	0.1625(37)	0.1779(38)
$\Delta_{K}\left(\times 10^{3}\right)$	1.81403(24)	1.894(173)	1.81403 ${}^{a}$
$\Delta_{JK}\left(\times 10^{3}\right)$	0.49995(32)	0.106(50)	0.49995 ${}^{a}$
$\delta_{J}\left(\times 10^{3}\right)$	0.0403044(13)	0.0403044 ${}^{a}$	0.0403044 ${}^{a}$
$\delta_{K}\left(\times 10^{3}\right)$	0.80893(11)	0.80893 ${}^{a}$	0.80893 ${}^{a}$
$\Phi_{J}\left(\times 10^{9}\right)$	0.02717(76)		
$\Phi_{JK}\left(\times 10^{9}\right)$	−0 .3231(55)		
$\Phi_{K}\left(\times 10^{9}\right)$	−2 .2598(265)		
IR RMS	0.0125	12.6	12.9
N lines	2488	624	244
$J^{\prime \prime }$	1–89	1–42	5–46
$K_{a}^{\prime \prime }$	0–38	0–18	0–12

${}^{a}$ Fixed to the GS value.

**Table 4:** Deviation (δ = exp-calc) of the rotational constants in the ground and excited states of *trans*-2-FF and *cis*-2-FF. All values are in MHz. For each state, we calculated the second (or planar) moment $M_{cc}$ in $amu-{\overset{˚}{A}}^{2}$, defined from the principal inertia moments by $M_{cc}=\frac{I_{a}+I_{b}-I_{c}}{2}$.

Trans-Furfural					
		Calculated	Experimental	δ = exp-calc	δ corrected
					from GS deviation ${}^{a}$
GS	A	8175.217	8191.774	016.557	
	B	2049.726	2045.930	−3.796	
	C	1638.853	1637.184	−1.669	
	$M_{cc}$	0.002	0.011	00.009	
$\nu_{17}$	A	8186.172	8196.994	010.822	−5.758
	B	2049.251	2045.282	−3.968	−0.173
	C	1637.714	1636.216	−1.499	00.169
	$M_{cc}$	−0.118	−0.061	00.057	00.052
$\nu_{23}$	A	8161.189	8178.013	016.824	00.295
	B	2049.062	2045.341	−3.722	00.074
	C	1639.227	1637.524	−1.703	−0.034
	$M_{cc}$	0.130	0.131	00.001	−0.008
$\nu_{14}$	A	8185.472	8196.395	010.923	−5.655
	B	2050.262	2045.341	−4.921	−1.123
	C	1638.627	1637.524	−1.103	00.565
	$M_{cc}$	−0.090	0.061	00.151	00.142
$\nu_{7}$	A	8162.687	8168.443	05.756	−10.776
	B	2048.163	2041.993	−6.170	−2.377
	C	1637.428	1636.883	−0.545	01.122
	$M_{cc}$	0.009	0.309	00.300	00.290
$\nu_{6}$	A	8158.790	8184.040	025.250	08.726
	B	2047.264	2044.086	−3.177	00.614
	C	1637.128	1635.632	−1.496	00.170
	$M_{cc}$	0.050	0.005	−0.045	−0.060
$\nu_{17}+\nu_{15}$	A	8191.921	8181.784	−10.137	−26.729
	B	2048.112	2043.667	−4.445	−0.652
	C	1635.617	1635.504	−0.113	01.553
	$M_{cc}$	−0.269	0.027	00.296	00.264
$\nu_{5}$	A	8165.685	8179.214	013.529	−3.009
	B	2047.563	2043.898	−3.666	00.127
	C	1637.128	1635.504	−1.624	00.043
	$M_{cc}$	0.006	0.023	00.017	00.008
**Cis-Furfural**					
GS	A	8139.736	8143.739	04.002	
	B	2101.222	2098.724	−2.497	
	C	1670.151	1668.873	−1.278	
	$M_{cc}$	0.005	0.017	00.012	
$\nu_{14}$	A	8149.311	8149.490	00.179	−3.829
	B	2101.883	2099.346	−2.537	−0.039
	C	1670.162	1668.998	−1.164	00.113
	$M_{cc}$	−0.068	−0.029	00.039	00.027
$\nu_{5}$	A	8131.539	8135.273	03.734	−0.265
	B	2099.061	2096.771	−2.290	00.205
	C	1668.441	1667.402	−1.039	00.237
	$M_{cc}$	0.005	0.028	00.023	00.010

${}^{a}$ Corrected values correspond to calculated ES constants corrected from the GS deviation, see Equation (2).

**Table 5:** Comparison between HITRAN vibrational integrated cross sections of 2-*trans*-FF measured at room temperature and those simulated from the molecular parameters determined in this work by jet-cooled high-resolution mid-IR rovibrational spectroscopy.

Wavenumber	Calculated from Our Simulations ${}^{a}$		Calculated from HITRAN ${}^{a}$
	**Without Hot Bands**	**With Hot Bands**	
**cm${}^{-1}$**	**cm.molecule${}^{-1}$**	**cm.molecule${}^{-1}$**	**cm.molecule${}^{-1}$**
700–795	$1.26\times 10^{-17}$	$2.23\times 10^{-17}$	$2.22\times 10^{-17}$
980–1050	$3.19\times 10^{-18}$	$5.89\times 10^{-18}$	$6.15\times 10^{-18}$
1450–1550	$4.5\times 10^{-18}$	$8.7\times 10^{-18}$	$1.17\times 10^{-17}$
1550–1610	$2.55\times 10^{-18}$	$7.67\times 10^{-18}$	$8.53\times 10^{-18}$
1660–1800	$1.29\times 10^{-17}$	$3.48\times 10^{-17}$	$3.75\times 10^{-17}$

${}^{a}$ Results obtained from integration of the cross sections given in cm${}^{2}$ molecule${}^{-1}$.

**Table 6:** Three experimental conditions used in this work with the Jet-AILES setup.

	Resolution	Optical Filter	Number of	Ar Flow	FF Flow	P${}_{\mathrm{stagnation}}$	P${}_{\mathrm{residual}}$	Slit Width
		Bandwidth	Averaged Scans					
	cm${}^{-1}$	cm${}^{-1}$		slm	slm	hPa	hPa	μm
$1{\;}^{a}$	0.55	None	30/30/30	5	0.5	95/123/252	0.23	100/80/50
$2{\;}^{b}$	0.00102	650–950	84	10	1.0	97	0.37	130
$3{\;}^{c}$	0.00102	1200–1800	160	10	1.0	320	0.37	50

${}^{a}$ Condition 1 refer to Fihure Figure 2; ${}^{b}$ Condition 2 refer to Fihure Figure 3; ${}^{c}$ Condition 3 refer to Fihures Figure 4 and Figure 6.

## Data Availability

The data presented in this study is available in the Supplementary Materials.

## Supplementary Materials

The following supporting information can be downloaded at: https://www.mdpi.com/article/10.3390/molecules28104165/s1, the compressed file SuppMat _furfural.rar contains the full list of GS rotational and ES rovibrational lines globally fitted of *trans*-2-FF (Table S1) and *cis*-2-FF (Table S2) as well as a series of graphs per rotational branch of observed-minus-calculated errors distribution as a function of K${}_{a}$ for the seven vibrational states of *trans*-2-FF analyzed (Graph G1).

## Abbreviations

The following abbreviations are used in this manuscript:

AILES	Advanced Infrared Line Exploited for Spectroscopy
CBS	Complete Basis Set
CD	Centrifugal Distortion
CEM	Controlled Evaporation Mixer
DFT	Density Functional Theory
EC-QCL	External-Cavity Quantum Cascade Laser
ES	Excited State
FF	Furfural
FT	Fourier Transform
FTIR	Fourier Transform InfraRed
FTMW	Fourier Transform MicroWave
FWHM	Full Width at Half Maximum
GS	Ground State
HITRAN	High-Resolution Transmission
IR	InfraRed
ip	In-Plane
MAE	Mean Absolute Error
MP2	Møller-Plesset perturbation theory at 2^nd^ order
MW	MicroWave
oop	out-of-plane
PNNL	Pacific Northwest National Laboratory
PT	Piezoelectric Transducer
QCL	Quantum Cascade Laser
RMS	Root Mean Square
slm	Standard liter per minute
SNR	Signal-to-Noise Ratio
SOA	Secondary Organic Aerosol
SOLEIL	Source Optimisée de Lumière d’Énergie Intermédiaire du LURE
SPIRALES	SPectroscopie InfraRouge Accordable par Laser dans une Expansion Supersonique
VOC	Volatile Organic Compound
VUV	Visible-UltraViolet
