# Supplementary material for: Rovibrational Spectroscopy of Trans and Cis Conformers of 2-Furfural from High-Resolution Fourier Transform and QCL Infrared Measurements
Source: Molecules. 2023 May 18;28(10):4165. doi: 10.3390/molecules28104165 (PMC10224235; doi:10.3390/molecules28104165)
Supplement: Supplementary file 1 [file molecules-28-04165-s001.zip › Graph_G1_O-C_error_distribution_Trans-2-FF.pdf]

Obs-Calc Error(blended) distribution of the trans-furfural Excited state ν_{17}

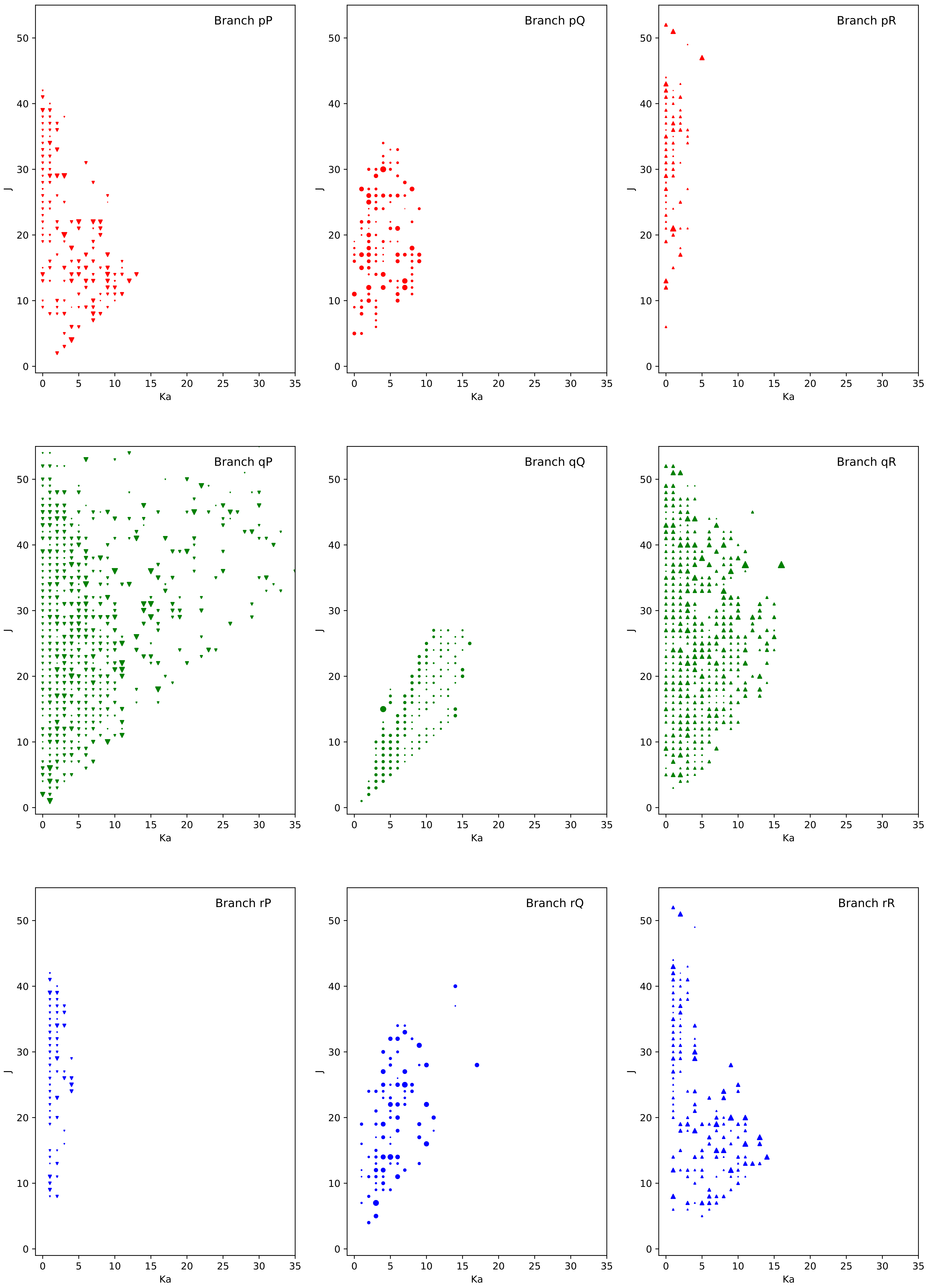

Obs-Calc Error(blended) distribution of the trans-furfural Excited state ν_{23}

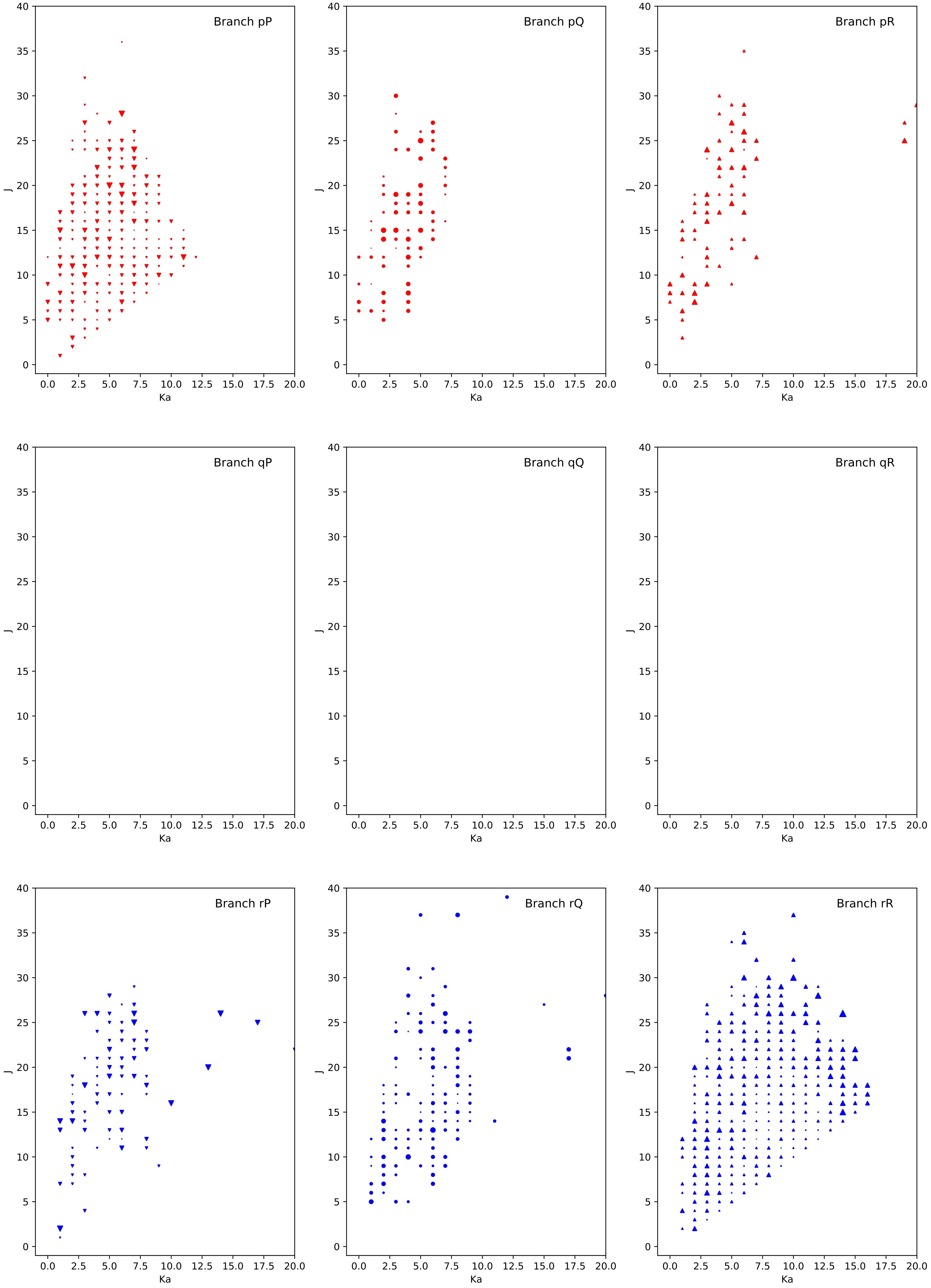

Obs-Calc Error(blended) distribution of the trans-furfural Excited state ν_{14}

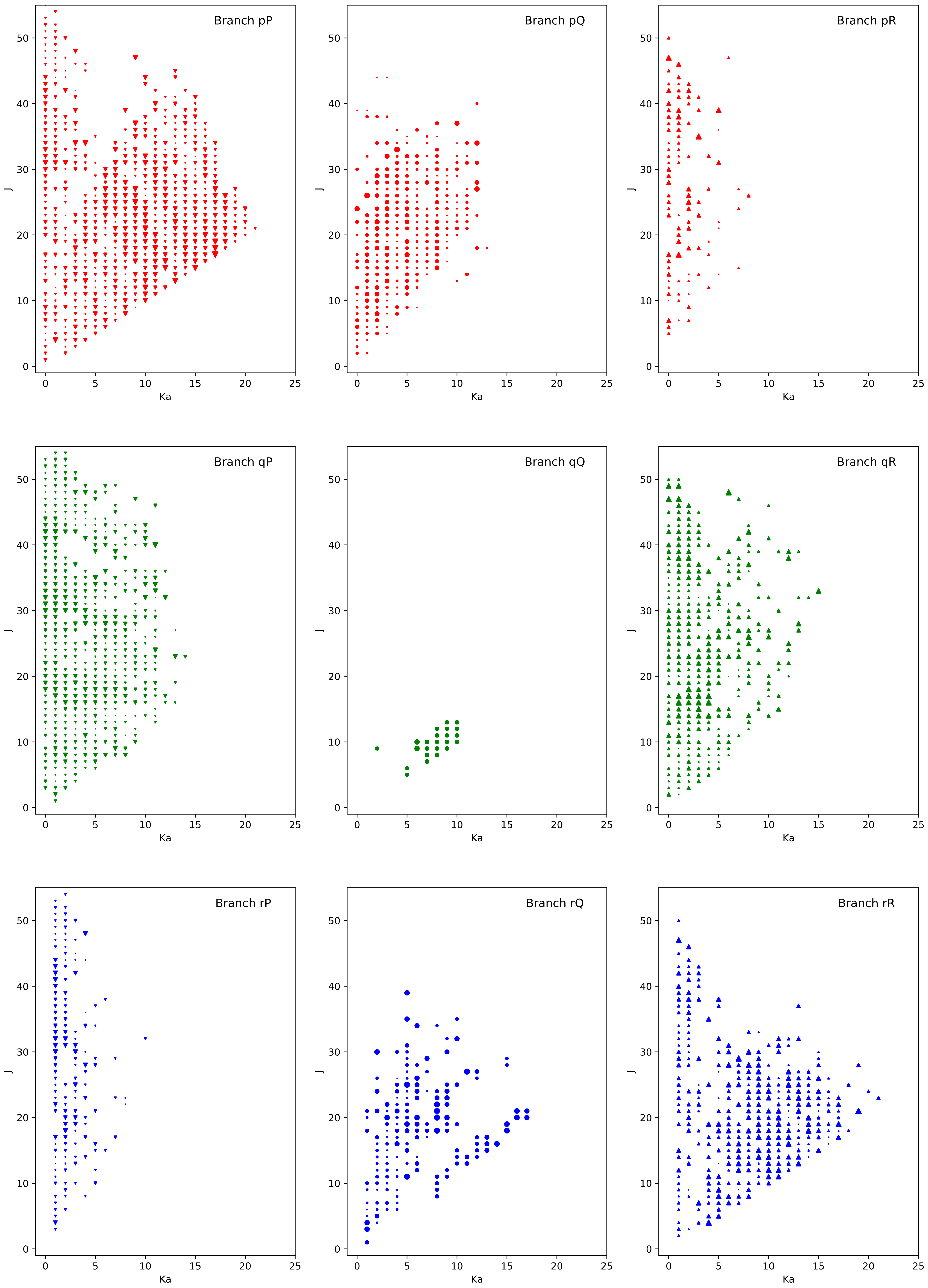

Obs-Calc Error(blended) distribution of the trans-furfural Excited state ν_7

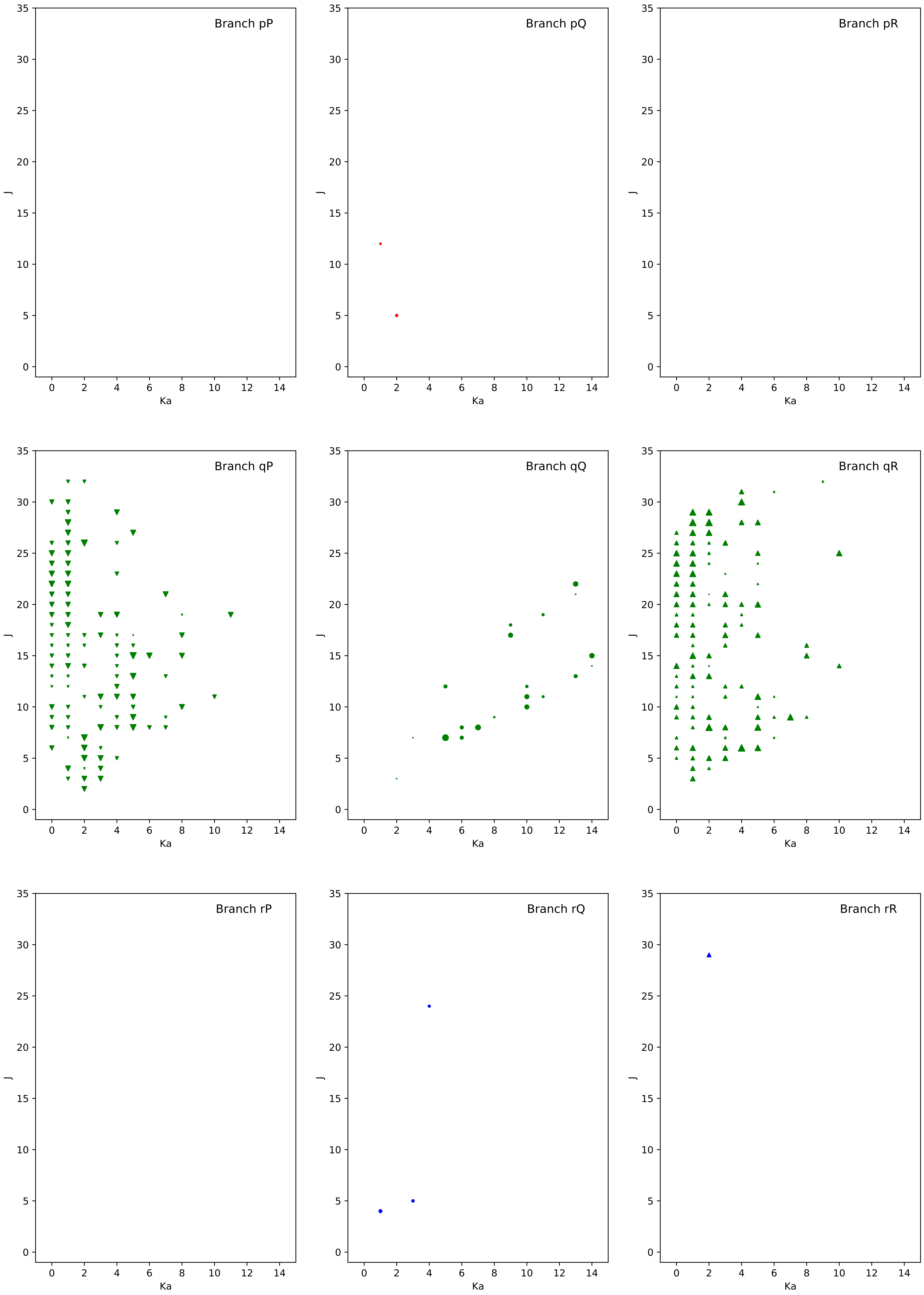

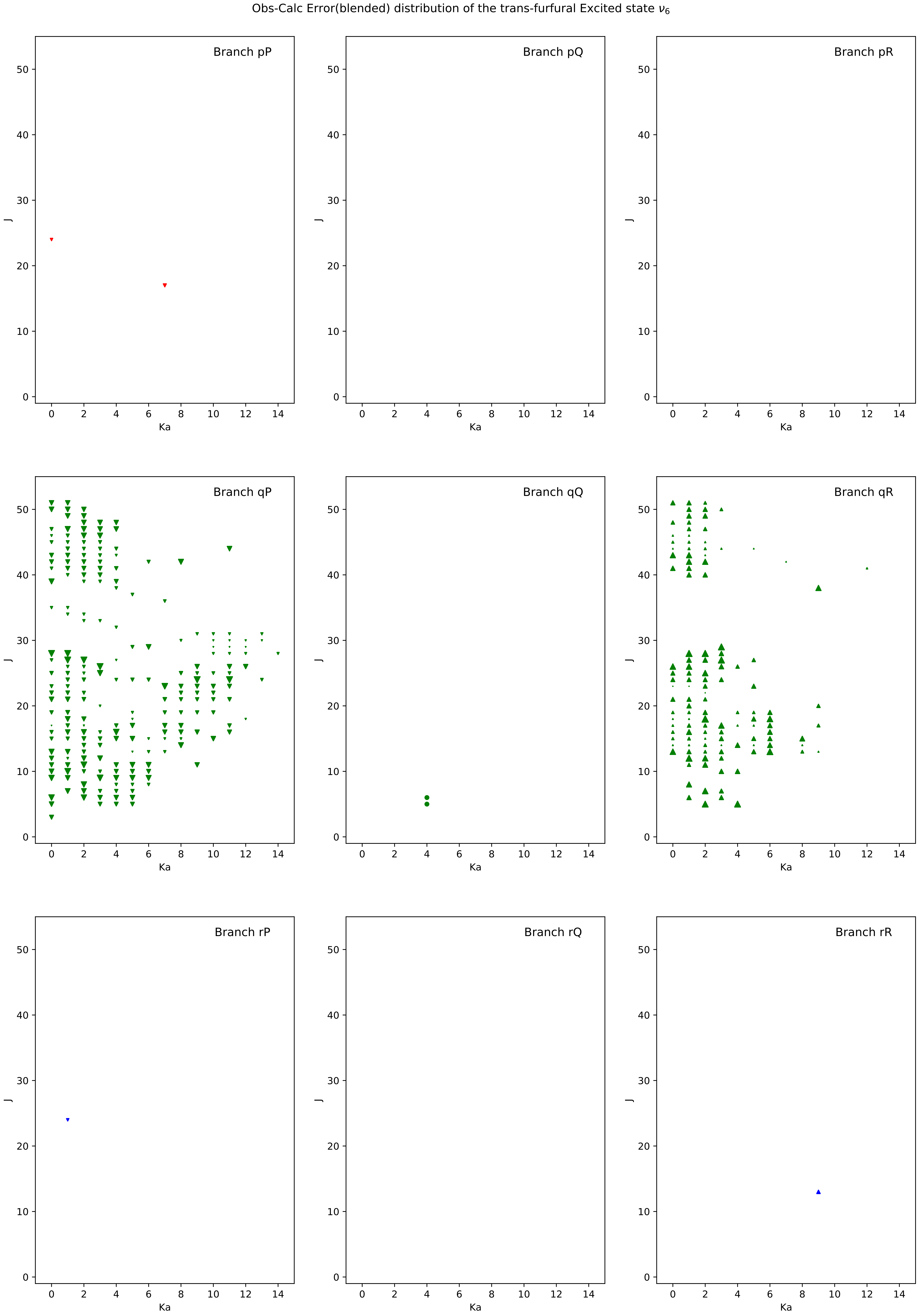

Obs-Calc Error(blended) distribution of the trans-furfural Excited state ν_6
Branch pP
Branch pQ
Branch pR
Branch qP
Branch qQ
Branch qR
Branch rP
Branch rQ
Branch rR
J
Ka

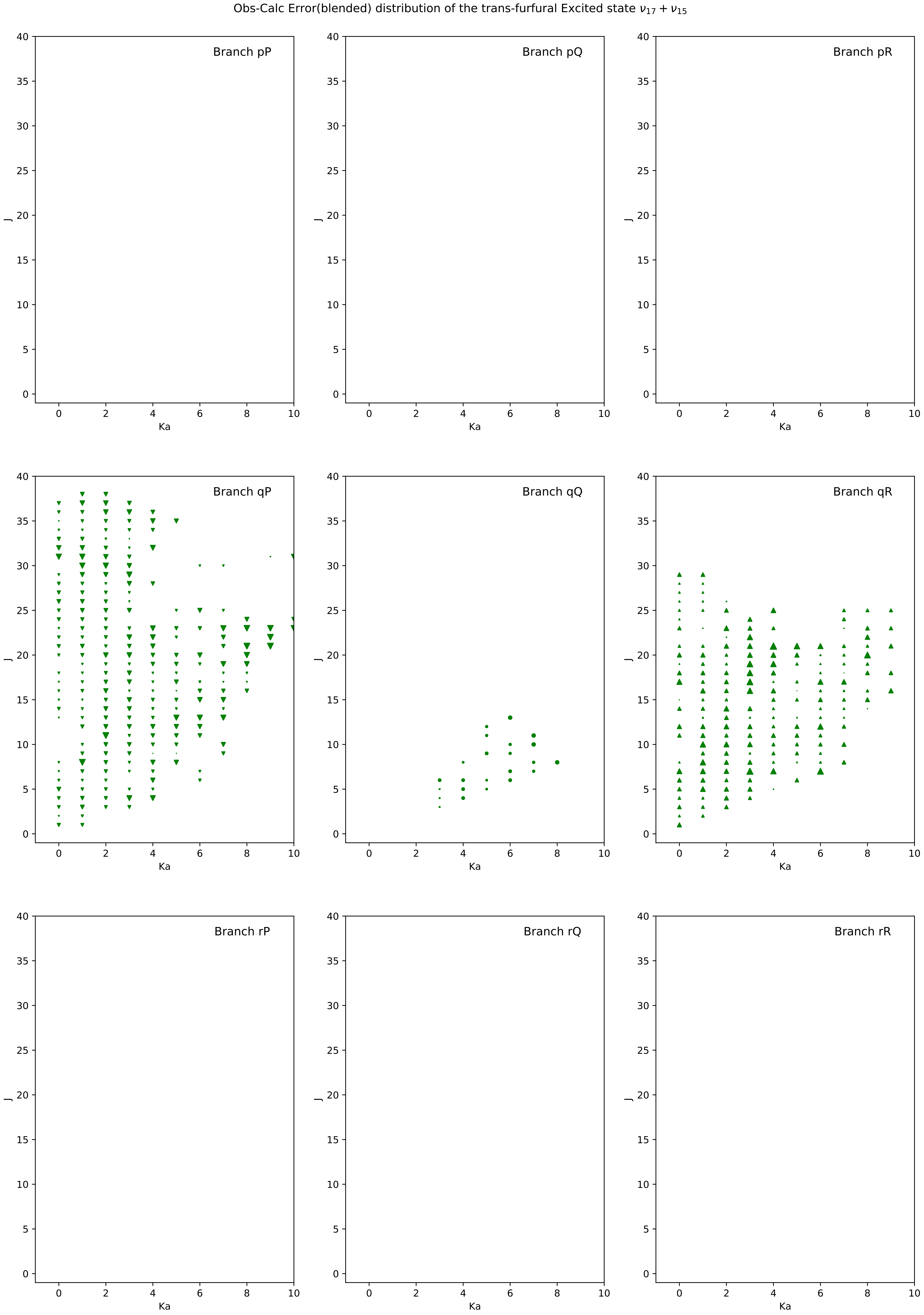
Obs-Calc Error(blended) distribution of the trans-furfural Excited state $\nu_{17}+\nu_{15}$
Branch pP
Branch pQ
Branch pR
Branch qP
Branch qQ
Branch qR
Branch rP
Branch rQ
Branch rR
J
Ka

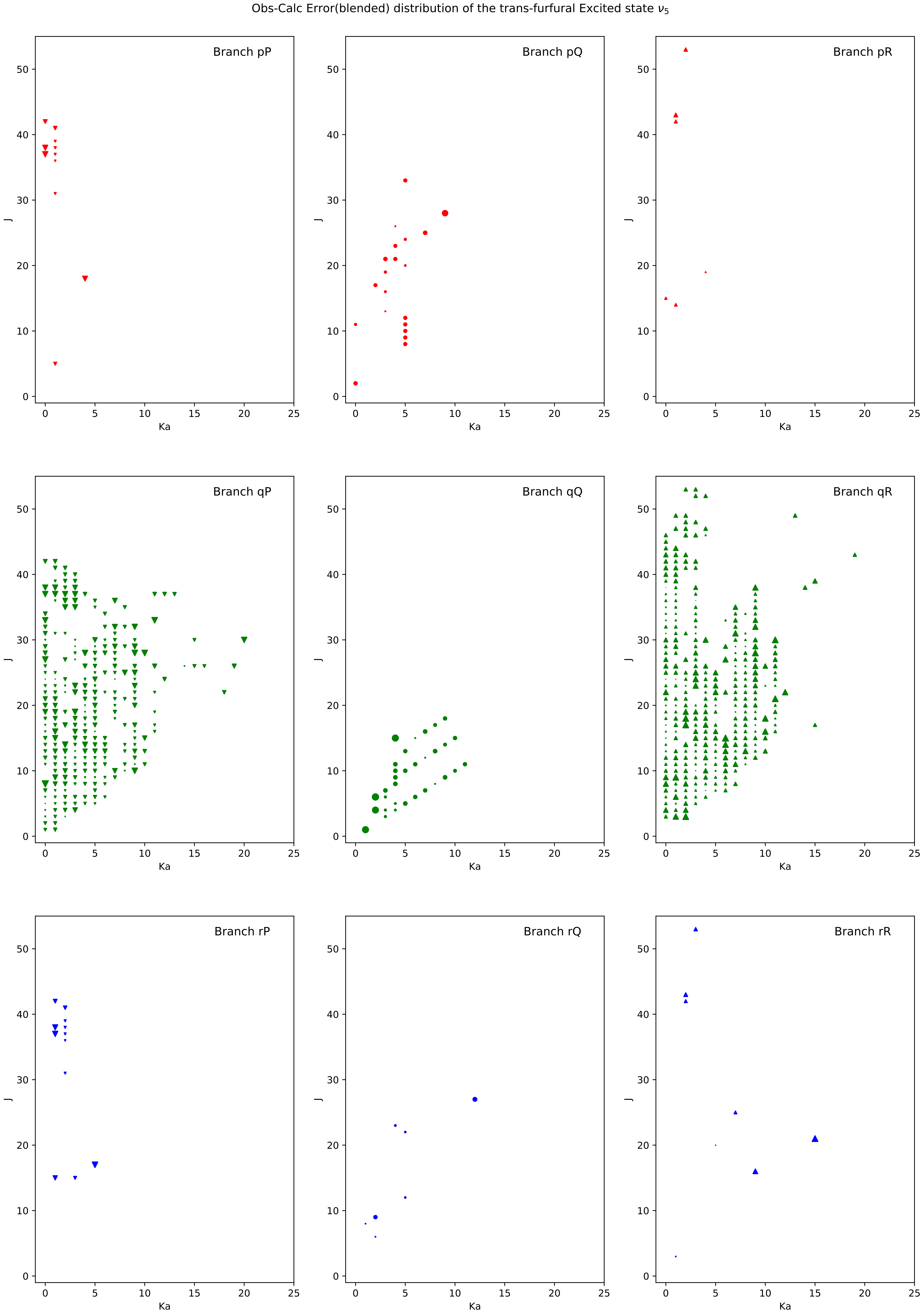
Obs-Calc Error(blended) distribution of the trans-furfural Excited state ν_5
Branch pP
Branch pQ
Branch pR
Branch qP
Branch qQ
Branch qR
Branch rP
Branch rQ
Branch rR
J
Ka
0
5
10
15
20
25
0
10
20
30
40
50
