# Supplementary material for: Rovibrational Spectroscopy of Trans and Cis Conformers of 2-Furfural from High-Resolution Fourier Transform and QCL Infrared Measurements
Source: Molecules. 2023 May 18;28(10):4165. doi: 10.3390/molecules28104165 (PMC10224235; doi:10.3390/molecules28104165)
Supplement: Supplementary file 1 [file molecules-28-04165-s001.zip › SuppMat-content.pdf]

Rovibrational spectroscopy of trans- and cis-conformers of 2-furfural from high resolution Fourier transform and QCL infrared measurements

Sathapana Chawananon, Pierre Asselin, Jordan A. Claus, Manuel Goubet, Anthony Roucou, Robert Georges, Joanna Sobczuk, Colwyn Bracquart and Arnaud Cuisset

The compressed file supp-mat-furfural.rar contains two tables in the .txt format and a pdf file containing a series of 7 graphs representing the distribution of observed-calculated (O-C) errors per rotational branch (p,q,r) for delta(K_a)= -1, 0, 1 and P,Q,R for delta(J)= -1, 0, 1) as a function of K_a of all rovibrational lines of *trans*-2-FF assigned in the vibrational states $v_{17} = 1$, $v_{23} = 1$, $v_{14} = 1$, $v_7 = 1$, $v_6 = 1$, v_{17}, v_{15} = 1,1 and $v_5 = 1$.

filename	prefix	content
Table *Trans*-2-FF	S1	linelist of GS and ES lines of *Trans*-2-FF
Table *Cis*-2-FF	S2	linelist of GS and ES lines of *Cis*-2-FF
Graph O-C error distribution *Trans*-2-FF	G1	O-C error distribution of ES lines of *Trans*-2-FF
